# Aberrant Pigmentation in a Schooling Cownose Ray (*Rhinoptera bonasus*) in Chesapeake Bay, Virginia, USA

**DOI:** 10.1002/ece3.72890

**Published:** 2026-01-04

**Authors:** Morgan F. Bennett‐Smith, Taylor Griffith, Helena Janulis, Eloise B. Richardson, Stephen J. Tomasetti

**Affiliations:** ^1^ Department of Biology & Boston University Marine Program Boston University Boston Massachusetts USA; ^2^ Mission Blue Napa California USA; ^3^ The Explorers Club New York New York USA; ^4^ Division of Biological and Environmental Science and Engineering (BESE) King Abdullah University of Science and Technology Thuwal Saudi Arabia; ^5^ Department of Natural Sciences University of Maryland Eastern Shore Sarbanes Coastal Ecology Center Berlin Maryland USA

**Keywords:** Batoidea, coastal ecology, color aberration, elasmobranchs, hypopigmentation, phenotypic variation, piebaldism

## Abstract

While pigmentation disorders such as albinism have been documented in a range of elasmobranch species, including the American cownose ray ( *Rhinoptera bonasus* ), the implications of these rare conditions for behavior, social dynamics, and fitness remain speculative and unverified. Here, we report a case of aberrant pigmentation in a single cownose ray observed schooling among conspecifics in the nearshore waters of Chesapeake Bay, Virginia, USA. The individual displayed pronounced white coloration against a notably dark dorsal surface of both pectoral fins, contrasting sharply with the otherwise uniform brown tones of the surrounding rays. The ray was fully integrated into the school and exhibited no abnormal behavior, suggesting that this pigmentation anomaly did not disrupt social dynamics. This observation augments the small but growing number of reports of pigmentation disorders in *R. bonasus* , suggesting that important social acceptances required for incorporation into the aggregation were unimpeded by the aberrant pigmentation.

## Graphical abstract

We report a rare case of aberrant pigmentation in a cownose ray (*Rhinoptera bonasus*) observed schooling naturally within Chesapeake Bay, Virginia. Drone and in‐water imagery revealed a piebald‐like pattern without any disruption to coordinated group behavior, providing the first in situ evidence that pigmentation anomalies in this species do not necessarily impair social integration. This observation contributes to the growing record of phenotypic variation in elasmobranchs and highlights the importance of behavioral context in assessing potential fitness consequences of pigment disorders.

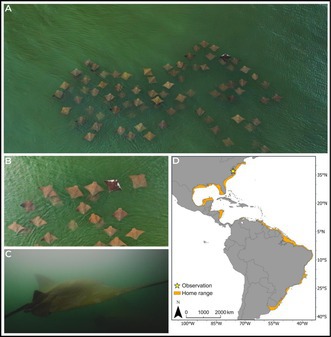

## 1 Introduction

Hypopigmentation disorders in marine fishes, including elasmobranchs, have been recorded across diverse species and environments, often as rare but visually striking anomalies (e.g., Shipley et al. 2022; Becker et al. 2023). Collectively, these disorders, which may be expressed as an absence of pigmentation (albinism) or a reduction in pigmentation (leucism), can result from genetic mutations, injuries, or environmental factors affecting pigment cell function (Abreu et al. 2013). Recent studies have begun to fill gaps in our understanding of their frequency among chondrichthyans, indicating a notable increase in reported sightings in recent decades, reflecting an increase in sampling opportunities, public engagement, and technological access (Quigley et al. 2018; Whitehead et al. 2025). Yet, the vast majority of documented cases in batoid species are limited to individuals that were captured, either during trawl surveys (Ball et al. 2013; Bigman et al. 2016; Lipej et al. 2011; Quigley et al. 2018) or as incidental bycatch (Rodrigues et al. 2023), rather than within their natural ecological context. One such study reported leucistic and potentially albino cownose rays, *Rhinoptera bonasus*, in the western South Atlantic, providing a recent confirmed record for the species (Rodrigues et al. 2023) to contrast with other reported observations made over half a century ago in the Chesapeake Bay (Schwartz 1959; Joseph 1961). These accounts exclusively described individuals that were captured in nets, and elasmobranch reports are generally based on single observations lacking behavioral context (Arronte et al. 2022; Clark 2002; Ratão et al. 2023; Skelton et al. 2024). Hypopigmentation may lead animals to incur fitness costs, including increased visibility to predators, altered thermal regulation, or impaired social interactions, as documented in other taxonomic groups (Dubovskiy et al. 2013; Slavík et al. 2015; Rose et al. 2017; Delhey et al. 2023). Yet very few observations documented wild behavior of pigment‐anomalous individuals, particularly in social, schooling species. Here, we contribute a novel in situ observation of aberrant pigmentation in a schooling cownose ray from Chesapeake Bay, Virginia, USA.

## 2 Observation

**FIGURE 1:**
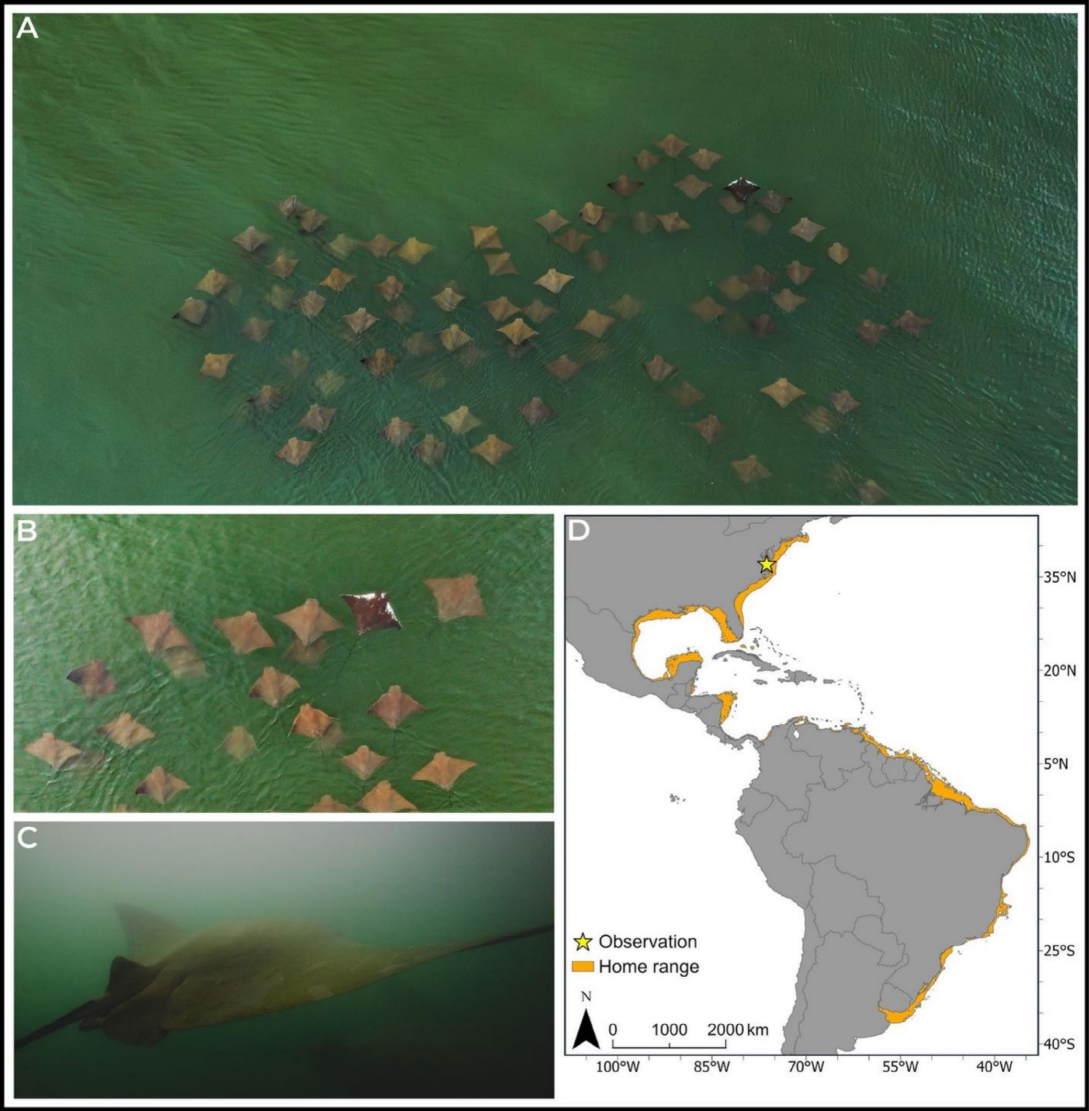
Observations of the schooling *Rhinoptera bonasus* , including (A) an aerial image of the aberrant ray integrated within the aggregation, (B) a closer view of the anomalously pigmented individual, (C) an in‐water screengrab of a typically pigmented individual from the aggregation, and (D) the locations of observations made for this species, across its biorange.

On May 16, 2025, during a nearshore aerial survey of marine fauna conducted as part of an Explorers Club field expedition, we observed and photographed a group of approximately 70+ cownose rays swimming in a tight school at the surface in shallow waters (~1.5 m depth) off the Virginia coastline of Chesapeake Bay near Gwynn's Island (37.0794 N, −76.2656 W). Among the group, one individual was visually distinct with white pigmentation extending across the anterior dorsal surface of both pectoral fins (Figure 1). The coloration on the rest of the dorsal surface appeared black (Figure 1). This pattern contrasted starkly with the uniform golden‐brown dorsal coloration typical of cownose rays, and observed in the rest of the school. The anomalously pigmented individual maintained coordinated movement with the group and exhibited no signs of stress or exclusion. The observation was recorded with a DJI Mavic 3 drone within local drone guidelines. Separately, in‐water observations of typically pigmented individuals from the same aggregation were made via snorkeling and captured with a Canon R5 inside a Nauticam underwater housing.

## 3 Discussion

Our rare imagery shows evidence of typical transiting behavior, and inclusion of a pigment‐anomalous batoid within a school of conspecifics in the Chesapeake Bay, a known hub for summer spawning activity. The pigmentation disorder observed likely represents a case of leucism rather than albinism, given the incidence of some coloration (Whitehead et al. 2025). A variation of leucism, known as piebaldism, can result in lighter colored patches against a pigmented box, creating distinct patterns. This has been reported in 17 species of shark (Whitehead et al. 2025), with most batoid pigment disorders reported as albinism (Ball et al. 2013; Bigman et al. 2016; Lipej et al. 2011; Rodrigues et al. 2023). Only a few reports allude to piebaldism in batoid species (Capapé et al. 2020, 2022). The pigmentation pattern in this individual—localized, symmetrical, and persistent—suggests a genetic or developmental origin rather than injury‐related scarring or epibiotic growth. Importantly, the integration of the pigmented individual into a normal schooling group supports the notion that such anomalies do not necessarily impair social behavior, although their influence on sexual selection or reproductive success remains unknown.

While previous reports, including Rodrigues et al. (2023), focused on pigment anomalies in cownose rays, they did not document social context or behavioral implications. Our observation offers rare visual evidence—including nearly 3 min of video footage showing coordinated movement (Video S1)—that the individual was not socially excluded, despite its striking coloration. Comparable reports are only sparsely supported by existing literature (Shipley et al. 2022; Ratão et al. 2023; Whitehead et al. 2025). Recent studies of nurse sharks reported normal swimming behavior of a solitary individual exhibiting piebaldism (Shipley et al. 2022; Becker et al. 2023) and a few occurrences where individuals with abnormal pigmentation were observed swimming with conspecifics, but neither study analyzed social integration in detail. In contrast, experiments with bony fishes described ostracism of anomalous individuals (Slavík et al. 2015), but this phenomenon remains unexplored in elasmobranchs. The existing literature suggests that our observation is one of a very small number of documented cases of a pigment‐anomalous elasmobranch exhibiting typical social behavior in the wild. However, further genetic and behavioral studies are needed to better understand pigment anomalies in schooling elasmobranchs.

Pigmentation anomalies in wild cownose rays are rare but increasingly documented. Observations similar to ours contribute to a growing baseline of phenotypic variation in marine elasmobranchs and may help inform future studies on the genetic, ecological, and evolutionary implications of pigment loss. The clear documentation of typical group behavior in a pigment‐anomalous individual may be particularly relevant to future assessments of fitness and social costs in wild populations.

## Author Contributions

**Morgan F. Bennett‐Smith:** conceptualization (lead), investigation (equal), methodology (equal), writing – original draft (equal), writing – review and editing (equal). **Taylor Griffith:** investigation (equal), writing – original draft (equal), writing – review and editing (equal). **Helena Janulis:** funding acquisition (equal), writing – original draft (equal), writing – review and editing (equal). **Eloise B. Richardson:** investigation (equal), writing – original draft (equal), writing – review and editing (equal). **Stephen J. Tomasetti:** funding acquisition (equal), supervision (lead), writing – original draft (equal), writing – review and editing (equal).

## Funding

This work was supported by Explorers Club.

## Conflicts of Interest

The authors declare no conflicts of interest.

## Supporting information


**Video S1:** Aerial video footage of a schooling cownose ray ( *Rhinoptera bonasus* ) with aberrant pigmentation in Chesapeake Bay, Virginia.

## Data Availability

There is no additional data. All data is included in the manuscript except for the full drone video, which was provided as a Supporting Information.
